# Polysaccharides as Effective and Environmentally Friendly Inhibitors of Scale Deposition from Aqueous Solutions in Technological Processes

**DOI:** 10.3390/polym15061478

**Published:** 2023-03-16

**Authors:** Alsu Venerovna Fakhreeva, Vasily Viktorovich Nosov, Alexander Iosifovich Voloshin, Vladimir Anatolyevich Dokichev

**Affiliations:** 1 Ufa Institute of Chemistry, Ufa Federal Research Center, Russian Academy of Sciences, Ufa 450054, Russia; 2 RN–BashNIPIneft LLC, Ufa 450103, Russia

**Keywords:** inhibitors of scale deposition, antiscalants, polysaccharides, flow assurance, environmentally friendly inhibitors

## Abstract

In this paper, we consider natural and modified polysaccharides for use as active ingredients in scale deposition inhibitors to prevent the formation of scale in oil production equipment, heat exchange equipment, and water supply systems. Modified and functionalized polysaccharides with a strong ability to inhibit the formation of deposits of typical scale, such as carbonates and sulfates of alkaline earth elements found in technological processes, are described. This review discusses the mechanisms of the inhibition of crystallization using polysaccharides, and the various methodological aspects of evaluating their effectiveness are considered. This review also provides information on the technological application of scale deposition inhibitors based on polysaccharides. Special attention is paid to the environmental aspect of the use of polysaccharides in industry as scale deposition inhibitors.

## 1. Introduction

Currently, the formation of deposits of poorly soluble salts from natural and technical aqueous solutions is a fundamental problem [1,2,3,4]. Natural water extracted from oil fields and process water used in technological systems are complex aqueous solutions that contain ions of salts of inorganic compounds. Under certain conditions, for example, a decrease in pressure or an increase in temperature, or when mixing different types of waters, dissolved ions are able to form poorly soluble salts, which, in turn, form mineral deposits. These processes are considered as an undesirable phenomenon, since they lead to the need for the repair and replacement of technological equipment and, accordingly, significant costs in the production of various products [1,2,3,4]. To ensure the scale stability of aqueous solutions, various inhibitors are used to prevent the crystallization of salts; they are considered to be the most effective at reducing the formation of scale deposits on the surface of equipment and, accordingly, can increase the rate of utilization and save energy. Inhibitors are considered a special class of chemicals that interfere with the crystallization process, slowing down or stopping the formation of poorly soluble salts in aqueous systems [1,2,3,4].

To date, various types of mineral deposits have been studied [5], and several methods for their prevention in technological processes based on scale formation inhibitors have been proposed [6,7]. The main mineral deposits that occur during oil production, in wells, pipelines, and in heat exchange equipment, are carbonates, sulfates, silicates, and calcium phosphates [4,8,9,10,11].

The problem of inhibiting scale deposits in technological systems is basically similar to preventing the formation of scale in washing machines; in both cases, similar chemicals are used. The inhibitor is added to an aqueous solution, in which it reacts through complexation with ions capable of forming a scale deposit. Conventional inhibitors of scale formation are hydrophilic and water-soluble. Scale formation inhibitors can be divided into two main groups: thermodynamic and kinetic. For example, substances that form complex or chelated compounds with a scale-forming cation belong to thermodynamic inhibitors. They are used to prevent deposits of specific salts, such as CaCO_3_, CaSO_4_, Fe(II), and Zn(II) sulfides [6]. The mechanism of action of kinetic inhibitors is usually considered in terms of stereospecific and nonspecific interactions [12]. The mechanism of inhibition of scale formation in the most general view can be described using adsorption effects [9,12] or morphological changes in crystal growth sites [12,13,14]. The adsorption effects are due to the fact that the inhibitor molecules occupy the sites of crystal nucleation and growth, thereby preventing the formation of crystal faces, or the inhibitor adsorption to the surface of the crystal embryo is considered, while further growth stops. Another mechanism of inhibition is based on the assumption that an inhibitor is introduced into the crystal structure while morphological changes in the crystal occur. Depending on the properties of the inhibitor molecule and the nature of the crystalline formation, it can be adsorbed onto the crystal lattice, forming complex surfaces. In this case, the crystal growth slows down significantly [14].

Modern scale formation inhibitors are able to prevent scale formation at a dosage of less than 30 mg/L. In this respect, polymer nitrogen compounds (polyacrylamide, hydrolyzed polyacrylamide, etc.), nonpolymer and polymer phosphorus compounds (phosphonates, aminophosphonates, and polyphosphinocarboxylates), and polycarboxylates (polyacrylate, copolymers of polyacrylate, and maleic anhydride) are very effective [3,4,12,13,14,15,16,17].

Approximately 5 × 10^6^ m^3^/day of oil is produced every day in the world, taking into account waterlogging and the need to protect equipment from scale deposition for approximately 10% of the extracted water; the amount of water that needs to be protected from scale deposition is approximately 10^6^ m^3^/day. This does not take into account water that is used in industrial systems and in heat exchange equipment.

Substances used to inhibit scale deposition can participate as nutrients in the process of eutrophication, which can lead to the mass development of some biological species and the death of aquatic organisms due to the deoxygenation of water [18]. Accordingly, over the past few years, industrial requirements for chemical compounds have concerned not only their effectiveness, but also their safety. The security requirements for these connections are as follows: they should not have mutagenic or carcinogenic properties and should also be less dangerous to the environment than the substances that are currently used [19]. Chemical emissions are strictly controlled through legislation, so it is important to find solutions that are environmentally friendly. In this regard, a scale deposition inhibitor can be defined as “green” in accordance with three criteria: nontoxicity, the absence of bioaccumulation, and rapid biodegradation in the environment [4,20]. In recent years, much attention has been paid in the literature to the development of new scale deposition inhibitors that are more environmentally friendly compared with conventional inhibitors [21]. Among the inhibitors of scale deposition, polysaccharides are perhaps the safest substances that are used in the industry. Moreover, polysaccharides in living organisms participate in and control biopolymerization processes [22,23,24,25,26,27,28]. This fact allows us to consider polysaccharides offered by nature as an inexhaustible renewable source for the construction of scale inhibitor molecules. The history of the use of inhibitors in technological processes began in the middle of the 19th century. However, the extracts of some plants were used in ancient Rome to prevent deposits of carbonate scale in aqueducts and canals [29]. In the 1930s, “calgon glass” (1.1 mol Na_2_O: 1 mol P_2_O_5_) was the first commercially produced scale inhibitor as a substitute for Na_3_(PO_4_)_2_. In the early 1940s, it was found that inorganic polyphosphates were effective in inhibiting calcium carbonate precipitation in concentrations of only a few parts per million doses, far below the stoichiometric quantity. This type of treatment is known as the threshold treatment. During the early 1950s, most of the focus on scale control was still on the internal treatment of boilers; in particular, the use of low-molecular polyacrylic acid began, which replaced natural tannins and lignins in the conditioning of boiler water sludge. The decade of the 1960s was marked by significant advances in the technology of inhibiting the formation of scale. Synthetic dispersants and inhibitors, such as polyacrylates and polymethacrylates, were used to inhibit scale deposits. The use of synthetic dispersants has significantly improved the prevention and removal of deposits caused by iron oxide, hydroxides, calcium phosphate, and silicon dioxide. While inorganic polyphosphates have been gaining popularity as a type of scale formation inhibitor since the 1930s, another class of water-softening compounds based on the use of phosphonates in cooling water recirculation systems, boilers, desalination technologies, and oil well systems was also developed in the 1960s and 1970s [29].

Tannins, lignin, starch, and plant resin (acacia Catechu) were used as inhibitors [30]. The use of plant extracts is currently considered as a promising method for preventing scale deposition [31]. Indeed, plant extracts are an interesting alternative source of organic molecules, mainly polysaccharides, since they are harmless to the environment, can be easily extracted, and the raw materials for their production are renewable [32,33].

This review summarizes the results of studies on the effect of polysaccharides, when considered as inhibitors, on the formation of poorly soluble salts from supersaturated aqueous solutions, and provides information on methods for evaluating the effects of inhibition. The last few years’ worth of data on promising natural and functionalized polysaccharides for their use as environmentally friendly scale formation inhibitors in various fields, such as oil production, energy, water supply, and food industries, are considered.

## 2. Composition and Saturation of Formation and Industrial Waters

The sources of water used in technological processes are surface water, underground water, and marine water. Any water from these sources is characterized by a variety of parameters, some of which are due to the nature of its genesis [1,34,35]. The most important parameters are the ionic composition and the number of dissolved gases within the water, primarily CO_2_ and H_2_S. The composition of natural compounds dissolved in water is determined through the composition of the lithosphere and the prevalence of its individual constituent elements. The prevalence and content of mineral substances in natural waters are different, with macro- and microcomponents distinguished among them [36]. Macrocomponents determine the chemical type of water, its total mineralization (TDSs—total dissolved solids), and the name of the total chemical composition. The main macrocomponents are the most common cationic (Ca, Mg, Na, K, and Fe) and anionic (Cl, S, C, Si, and O) elements. The increase in the mineralization of natural waters occurs due to the appearance of soluble compounds in solutions. The most mineralized (TDS ≈ 170–360 mg/L) are high-salinity calcium chloride brines, and the least (TDS < 1 g/L) are sweet calcium bicarbonate and calcium hydrosilicate waters [34]. Microcomponents are contained in natural water, as a rule, in insignificant quantities, defined as quantities of milligrams, micrograms, or less in 1 L of water. Ultimately, only a relatively small number of ions are present as the main constituents in almost all natural waters; these are Na^+^, K^+^, Ca^2+^, Mg^2+^, Sr^2+^, and Ba^2+^ cations and Cl^−^, CO_3_^2−^, and/or HCO_3_^–^ and SO_4_^2–^ anions. Acidic natural waters contain Fe^2+^, Mn^2+^, borate, NH_4_^+^, Br^−^, and I^−^ ions, and Fe^3+^ surface water. Unusual associates and ionic complexes are found in highly concentrated mixed salt solutions [37]. Ionic complexes can be ionic pairs in which the cation retains its hydrate shell unchanged. The bonds between ions can be formed through an electrostatic interaction or a donor–acceptor mechanism, forming coordination complexes in which one or more water molecules are bound to a cation through covalent bonds. Metal ions of s- and d-elements can form many specific complexes in highly concentrated salt solutions [38]. The structures of aqua-, carbonate-, sulfate-, and chlorometallic complexes in highly concentrated mixed salt solutions at high pressures and elevated temperatures have been studied very little, and remain a complex field of scientific research. For example, when the pressure/temperature changes, depending on the pH of the solution, carbonate complexes transform into aquocomplexes with the increase in pH. Most common ions, including Cl^−^ and SO_4_^2−^ ions, form many ion pairs in high-salt solutions. Trace elements and low concentrations of other elements are mainly found in concentrated mixed-salt solutions in the form of coordination complexes, such as [Al(H_2_O)_6_]^3+^ and [Pb(H_2_O)_6_]^2+^, and in the form of soluble silicon complexes. It is also necessary to take into account the numerous associative compounds between Mg^2+^ and SO_4_^2−^ ions and unstable carbonate complexes, which decompose with decreases in pressure and oxygen access. In the presence of CO_2_ in systems with high salinity, well-known patterns of salt solubility are not applicable and, therefore, often lead to misinterpretation and incorrect estimates regarding precipitation and the sediments that arise during production processes. The following ion concentrations are present in natural water [34,39,40]:
>10,000 mg/LNa^+^, Cl^−^100–10,000 mg/LCa^2+^, Mg^2+^, K^+^, Sr^2+^, Ba^2+^, SO_4_^2−^1–100 mg/LAl^3+^, Fe^2+^, Li^+^, borates, silicates, NH_4_^+^, HCO_3_^−^/CO_3_^2−^<1 mg/LZn^2+^, Hg°, Be^2+^, Co^2+^, Pb^2+^

Thus, the presence of dissolved salts in natural water forces us to consider it as a physicochemical system—an electrolyte—in which the properties are determined with specific interactions between ions, forming a network of hydrogen bonds between water molecules and the structure of ion aggregation. It has always been assumed that the physical forces acting between ions and water molecules, as well as between ionic aggregates, should explain many of their physical and chemical properties. The basic idea representing the electrolyte as a system in the chemical equilibrium of free ions, ion pairs, and higher associates belongs to Arrhenius, who introduced the law of acting masses for chemical equilibrium between free ions and salt molecules. In this case, complete dissociation is implied in the limit of infinite dilution, and with the increase in the concentration of salts, a decrease in the degree of dissociation follows. The average size of large ion aggregates in aqueous solutions increases with an increasing ion concentration, and the largest aggregates can be considered as intermediate states connecting free ions with macroscopic aggregates, such as solid salts. It is possible to imagine the concept of the critical concentration of ion aggregation (CCA) for various spontaneously collecting systems preceding crystallization [41]. It was found that the appearance of large ionic clusters in supersaturated CaCO_3_ solutions is the key to understanding the beginning of mineralization, for example, in CaCO_3_ deposits [42].

The process of the deposition of inorganic salts is directly related to a significant oversaturation of the aquatic environment due to changes in the physicochemical parameters of oil production systems (temperature, pressure, gas release, concentration of sedimentary ions, etc.). The chemical composition of natural waters in technological processes, especially in oil production, constantly changes as oil reserves are developed, which causes diversity and variability in the composition of scale deposits over time [43,44,45,46]. The precipitation of a solid substance occurs if its concentration in the solution exceeds the limit solubility of the salt for these conditions. A poorly soluble salt in the solid state is in equilibrium with scale-forming ions in solution, according to the following equation:

*Kt*_m_*An*_n_ ↔ *m*·*Kt*^+^ + *n*·*An*^−^, (1)

The supersaturation degree of the *SR* solution can be determined with the following formula:

(2) $$SR=\frac{{\left[a_{Kt^{+}}\right]}^{m}\cdot {\left[a_{An^{-}}\right]}^{n}}{K_{sp}}$$

where $a_{Kt^{+}},a_{An^{-}}$- is the activity of the cation and anion, and *K_sp_* is the product of solubility, depending on the temperature and pressure.

The tendency of inorganic compounds to form insoluble precipitates can be determined with the value of *SR*; if *SR* > 1, the solution is supersaturated. For convenience, the saturation index *SI* can be used:

(3) $$SI=lg\frac{\left[a_{Kt^{+}}\right]\left[a_{An^{-}}\right]}{K_{sp}}$$

Accordingly, at *SI* > 0, salt precipitation is possible, whereas, at *SI* < 0, precipitation would not fall out, and the solution is able to additionally dissolve salt.

## 3. The Main Causes of Scale Deposits Formed in Technological Processes, Their Compositions, and Their Structures

An increase in the actual concentration of scale-forming ions and an excess of soluble products can be realized, for example, when mixing waters of different compositions that are incompatible with one another, as well as when dissolving rocks. A change in thermobaric conditions can also lead to a change in the solubility of the salt. In the technological chain of oil production, the shift in the ionic equilibrium towards sedimentation as a result of changes in temperature and pressure during the rise and movement of reservoir water is especially characteristic of the formation of CaCO_3_ deposits:

Ca^2+^ + 2HCO_3_^−^ ↔ CaCO_3_↓ + H_2_O + CO_2_↑ (4)

It is known that carbon dioxide exists in both free and dissolved forms in reservoir waters. It can be present in the form of undissociated molecules of carbonic acid, H_2_CO_3_, bicarbonate ions, HCO_3_^−^, and carbonate ions, CO_3_^2−^. In the presence of Ca^2+^ ions, the particles CaHCO_3_^+^, CaOH^+^, and CaCO_3_ form in solution, with association constants ${pK}_{CaHCO_{3}^{+}}$, ${pK}_{CaOH^{+}},\;{and\;pK}_{CaCO_{3}}$ of 1.26, 1.49, and 3.22, respectively [47,48].

The solubility (−lg(p*K*_sp_)) of various crystalline modifications of calcium carbonate and their hydrated forms is given in Table 1 [49,50,51]. The solubility of iron and magnesium carbonates is shown in the same table. The solubility of hydrated forms of CaCO_3_ is higher than that of their anhydrous forms of calcite, aragonite, and vaterite [49].

**Figure 1:**
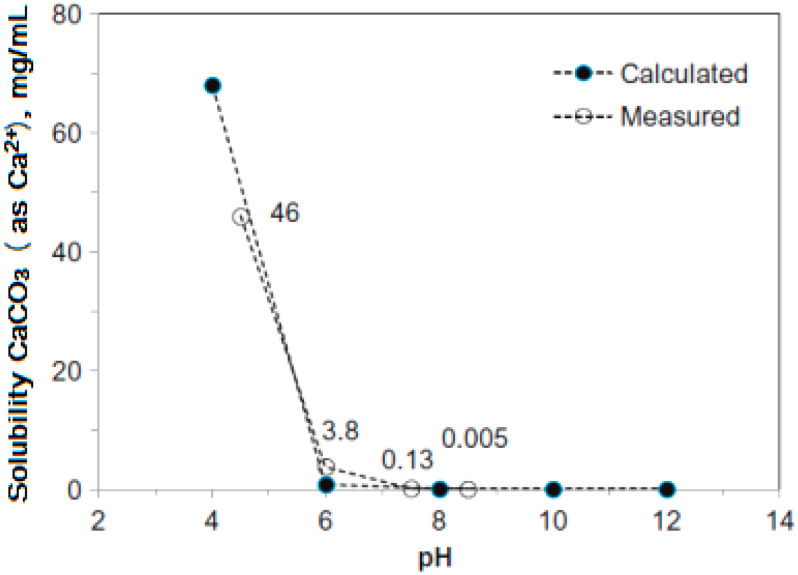
The calculated and measured solubility of CaCO_3_ as a function of pH of solution open to the atmosphere at room temperature.

The solubility of CaCO_3_ in water, depending on the pH, is shown in Figure 1.

In the presence of Ca^2+^, Mg^2+^, and HCO_3_ ions in reservoir waters at the same time, unstable soluble calcium and magnesium bicarbonates are formed, the equilibrium content of which is also maintained by free carbon dioxide.

When oil, gas, and reservoir water move along the borehole, discharge lines, and collectors, the pressure in them decreases, and the solubility of CO_2_ in water decreases, resulting in carbon dioxide being released, while the carbonate equilibrium is disturbed (Equation (4)). To restore equilibrium, the excess of bicarbonate ions, HCO_3_^−^, is removed from the system through the transformation of calcium and magnesium bicarbonates into carbonates that precipitate.

The influence of petroleum components on the process of scale deposition is considered in [52], in which the authors point to the important role assigned to water-soluble petroleum components; in particular, the role of naphthenic acids and their salts in the formation of scale deposits. The influence of oil components on the process of scale deposition is reduced to the hydrophobization of salt crystals formed in the flow volume due to the adsorption of water-soluble oil components, mainly petroleum acids and their salts. The adhesion of scale particles to one another and to the walls of pipes can be explained with the hydrophobicity of their surface. A significant part of precipitation contains both crystalline salts associated with naphthenic components and adsorbed organic compounds that hydrophobize the crystal surface [52].

It has been established that an increase in the number of organic components of oil in the solution entails an increase in the rate of sedimentation. Electron microscopic studies have shown that the main reason for this is the adsorption of organic compounds on the surface of scale particles [53].

In low-watered wells, a factor affecting scale deposition is the partial evaporation of water into the gas phase during the degassing of well products. During the evaporation of water, there is a general decrease in the solubility of salts. Salts that are soluble under normal conditions, including chlorides of alkaline and alkaline earth metals, such as halite [54,55,56,57], can pass into the precipitate.

Summarizing the information discussed in this section, the following main causes of scale formation could be identified:

1. Calcite precipitation occurs when thermobaric conditions change, mainly with a decrease in pressure and an increase in temperature. When the pressure decreases, carbon dioxide is released from the water, which leads to calcite precipitation. As a result, carbonates are deposited on the surface of pumping equipment and inside pumping and compressor pipes.
2. The mixing of incompatible waters leads to scale formation. When water containing calcium, barium, and strontium cations is mixed with water (for example, with sea water) containing sulfate ions, the poorly soluble minerals barite, celestine, gypsum, and anhydrite are formed.

Ba^2+^ (Sr^2+^, Ca^2+^) + SO_4_^2−^ → BaSO_4_↓ (SrSO_4_, CaSO_4_), (5)

When water containing hydrogen sulfide is mixed with fluids containing iron, zinc, or lead ions, sulfides are formed as deposits:

Zn^2+^(Pb^2+^, Fe^2+^) + H_2_S → ZnS↓(PbS↓, FeS↓) + 2H^+^ (6)

Moving through the reservoir during flooding, the injected water tends towards an equilibrium state with the rock at reservoir pressures and temperatures [36]. With a content of 0.2% carbonates and 0.4% sulfates in the rock, the equilibrium saturation of water with these ions occurs after 30 days. This leads to the composition of the extracted water at the deposit differing from the composition of the injected water, often being supersaturated by the main scale-forming ions.

3. A significant factor affecting scale deposition in low-watered wells is the partial evaporation of water into the gas phase during the degassing of borehole products. In the process of water evaporation, there is a general decrease in the solubility of salts, and even salts that are well soluble under normal conditions can enter the precipitate [55,56,57].
4. One of the reasons for the intensive deposition of calcium carbonate and gypsum in pumping equipment is the increase in the temperature of the flow of extracted products due to the friction of the pump impellers and heat transfer from a working submersible electric motor. An increase in the temperature due to the flow of the extracted products, depending on the flow rate of the well, occurs by 4–15 °C, which creates conditions for scale deposition on the wheels of the ESP [58].
5. When the well is put into operation after its silencing with scale solutions, intense scale formation is also possible. The reason for scale formation in this case is the increase in the concentration of scale-forming ions, decrease in the concentration of dissolved CO_2_, and change in the ionic strength of the extracted aqueous solutions. Favorable conditions for sedimentation can be realized when silencing with solutions of calcium chloride or other calcium-containing solutions. Sedimentation also occurs when wells are silenced with a sodium chloride solution, although less intensely [58].

In Table 2, the most typical mineral deposits found in the practice of oil production, their main crystalline forms, and sources of scale-forming ions are shown.

**Figure 2:**
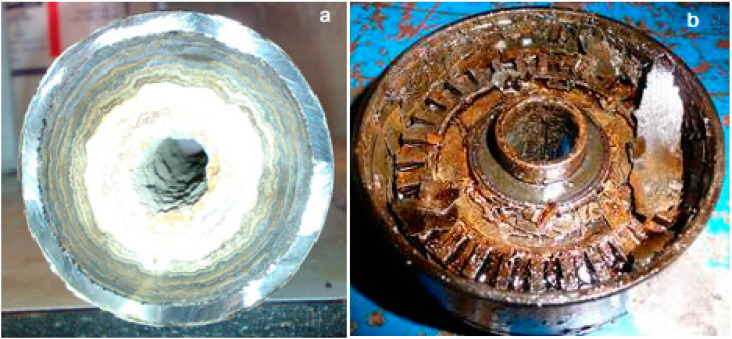
Calcium carbonate deposits in the pipeline (**a**) and the working organ of the submersible pump (**b**).

Scale deposits in oilfield practice, as a rule, are complex multicomponent formations, and are not monominerals. In addition to the mineral part, scale deposits include organic compounds such as asphaltenes, refractory paraffins, resins, bitumen, and sulfur compounds. The content of organic components can reach tens of percent [52]. According to the structure, the mineral part of scale deposits is represented by micro- and fine-crystalline sediments, dense layered structures with inclusions of organic components, which depend on the conditions of the deposits; an example of such deposits is shown in Figure 2.

**Figure 3:**
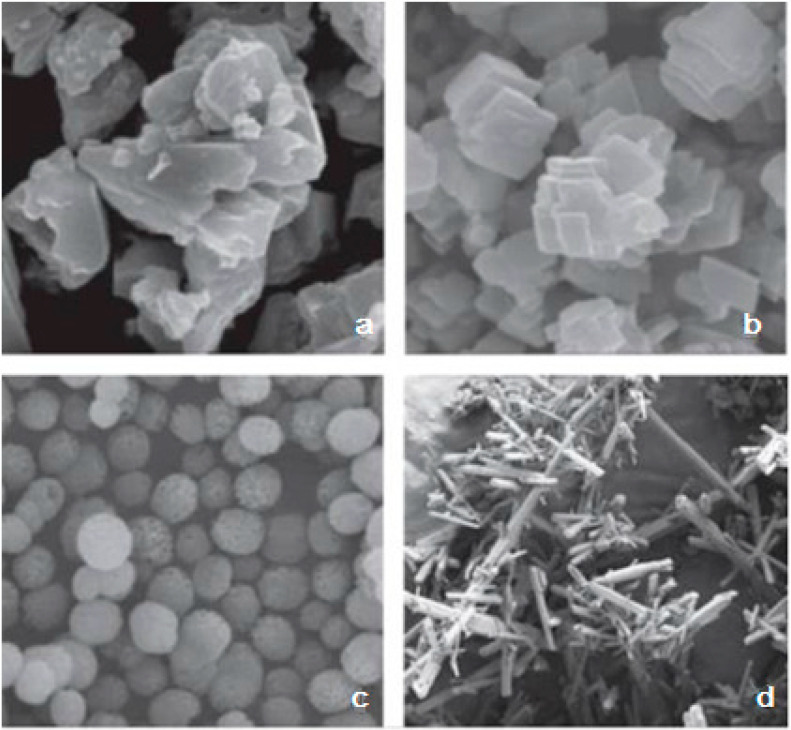
CaCO_3_ forms: amorphous calcite—(**a**); layered and rhombohedral calcite—(**b**); spherical vaterite—(**c**); needle-shaped aragonite—(**d**). Reproduced from [51], with permission from Elsevier, 2016.

**Figure 4:**
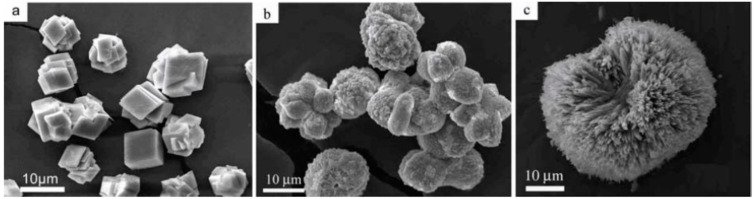
SEM images of CaCO_3_ crystals obtained at different ratios of [Mg^2+^]/[Ca^2+^] = 0 (**a**), 0.5 (**b**), and 3 (**c**); [CaCO_3_] = 8 mM. Reproduced from [65], with permission from Wiley, 2008.

The crystalline form of calcium carbonate in scale deposits is mainly represented by calcite, aragonite, and, to a lesser extent, vaterite [51,60,61], and the formation of a particular crystal structure strongly depends on the conditions under which crystallization occurs, the ionic composition of the water, and the impurities present (Figure 3). Thus, in the presence of diethylenetriaminepentaacetic acid (DTPA) at temperatures above 200 °C, the metastable form of CaCO_3_—vaterite—is mainly formed when CaCl_2_ and Na_2_CO_3_ solutions are mixed; at temperatures below 100 °C, both calcite and vaterite are formed [62]. It is obvious that DTPA interferes with the crystallization process by forming different CaCO_3_ polymopes. In [63], the rate of the transformation of polymorphic forms of calcium carbonate depending on temperature was considered, including in the presence of a phosphonate inhibitor. It was shown that calcite is the dominant form in the polymorph distribution without an inhibitor, with a crystallization time of less than 10 min at temperatures below 30 °C, calcite transforms into vaterite with the increase in temperature, and aragonite appears from 40 °C. All crystalline forms eventually turn into calcite at any temperature. The influence of alkaline earth metal ions present in the extracted water on the crystallization of calcium carbonate is of practical importance for understanding the mechanism of sediment formation. Thus, the influence of the Mg^2+^ ion concentration and temperature on the crystal structure of CaCO_3_ was shown in [64,65]. It was found that the composition of polymorphic calcium carbonate formed during precipitation was determined by both the pH value and the duration of the crystallization process [64]. At t = 80 °C in the presence of Mg^2+^ ions, the main polymorphic modifications of calcium carbonate were aragonite and magnesian calcite, and the content of aragonite increased as the duration of the crystallization process increased. Scanning electron microscopy (SEM) methods have recorded a strong influence of Mg^2+^ ions on the structure of CaCO_3_ crystals, and at certain ratios of [Mg^2+^]/[Ca^2+^], it was possible to achieve the formation of microspheres [65] (Figure 4).

**Figure 5:**
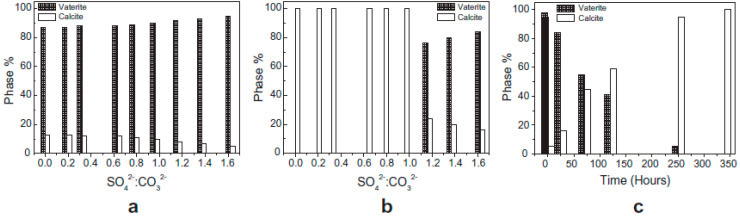
Distribution of calcite and vaterite depending on SO_4_^2−^/CO_3_^2−^ in the initial solution after 5 min (**a**) and after 24 h (**b**). Distribution of calcite and vaterite depending on the holding time of crystals in solution at an initial ratio of SO_4_^2−^/CO_3_^2−^ = 1.62 (**c**). Reproduced from [66], with permission from Elsevier, 2010.

The effect of sulfate ions on CaCO_3_ crystallization was investigated in [61,66]. It was shown that SO_4_^2−^ could stabilize the resulting vaterite, mainly formed during the first minutes of crystallization, at a ratio of SO_4_^2−^/CO_3_^2−^ < 1 after 24 h. Calcite is a single phase in the mixture, but at a ratio of SO_4_^2−^/CO_3_^2−^ > 1, vaterite can persist for a long time (Figure 5).

It is obvious that in the presence of impurities interacting with crystallizing particles, their involvement in the crystallization process inevitably occurs. Moreover, impurities can be added either to increase the crystallization rate, or to inhibit [67] or control crystal morphology [68,69]. The inclusion of such foreign substances can modify the crystallization pathways in various ways, such as by (1) changing the arrangement of the atomic and molecular structures of precursors (crystalline polymorphs) [70], (2) modulating the shape of crystals [69], and (3) directing and stabilizing the formation of nanoparticles at the nucleation stage [71], as well as when the crystal reaches large sizes to prevent or promote aggregation. In the literature, one can find several studies devoted to the study of the effect of additives on crystallization [72], in which models of crystal growth are proposed with the assumption that the crystal composition does not change during formation [72]. However, taking into account more recent studies of various intermediate precursor phases, it can be expected that additives can interact with them, potentially changing the crystallization pathways. It is obvious that additives, which can be polymer molecules, change not only the mechanisms of crystal growth [73,74], but also their thermodynamic state and crystallization kinetics related to the structure and composition of nucleation precursors during crystallization. The analysis of complex effects affecting the nucleation and growth of crystals and the role of additives during crystallization allows us to better understand the mechanisms of crystallization, clarifying some issues. Among these questions, we wished to highlight the following: (1) how crystallization occurs along the “nonclassical” pathway of nucleation controlled by additives [75], (2) how rare and unstable polymorphs arise during crystallization in the presence of polymers [76,77], and (3) how to control the final morphology of nanoscale crystals for various applications of interest [77,78].

## 4. Scale Inhibitors

Inhibitory methods of preventing scale deposition have been given priority in oilfield practice, water supply, and the protection of heat exchange equipment [1,2,3,4,79,80]. Substances that inhibit the crystallization of salts in technological systems can hardly be considered diverse. The main classes of scale inhibitors are based on their functional groups and molecular structures. Important classes of compounds include phosphonic acids, sulfonates, and carboxylates [15,17,81]. Phosphonates are generally used to inhibit various carbonate and sulfate deposits of alkaline earth metals. Deprotonated groups of phosphonic acid have a high affinity for alkaline earth ions [82] and crystal surfaces [83]. Carboxylates are typically used as scale deposition inhibitors, although to a lesser extent [84].

**Figure 6:**
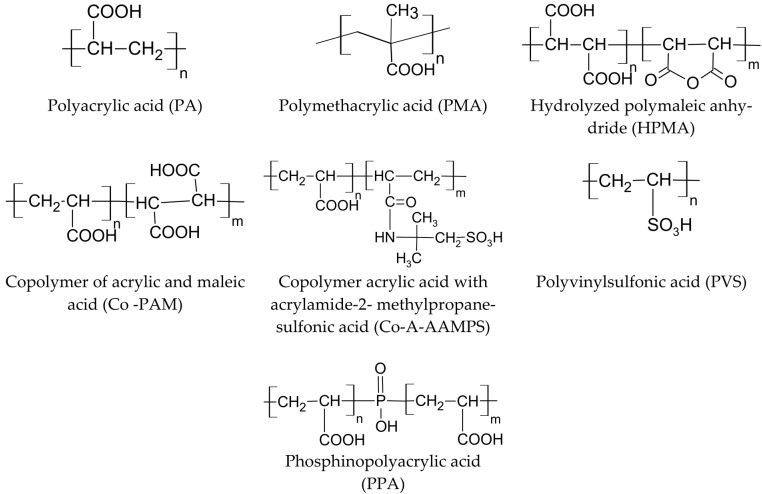
Examples of structures of anionic polymers used to inhibit carbonate and sulfate scales of alkaline earth metals.

Anionic polymers—polycarboxylates, for example—based on polyacrylate are widely used in various water-purification technologies [85]. Most anionic polymers are polyelectrolytes based on polyacrylate [86]. These can be polyacrylic acid homopolymers, copolymers containing a second functional group, such as sulfonate, or terpolymers containing a third functional group, such as phosphonate [87,88,89] (Figure 6).

**Figure 7:**
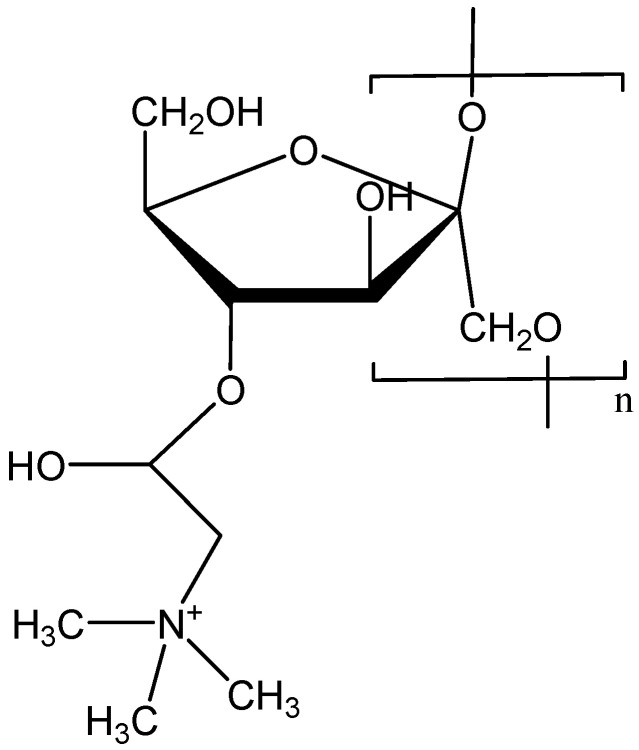
Cationic inulin.

Cationic polymers are specific inhibitors of the formation of silica deposits. Colloidal silicon dioxide is a polymer compound consisting of Si-O bonds. Consequently, the polymer inhibitor should be able to stabilize silicic acid Si(OH)_4_ or its deprotonated form Si(OH)_3_O^−^, that is, the precursor of silica deposition (Figure 7). Poly(1-vinylimidazole) [90], polyaminoamide dendrimers with an amine end (PAMAMs) [91,92,93,94], poly (acrylamide-co-diallyldimethylammonium chloride) [95], polyethylene amine (branched and linear) [96,97], and cationically modified inulin [98] have been shown to slow down the condensation of silicic acid at dosages of approximately 100 ppm in supersaturated solutions of silicic acid. It is assumed that the positive charge on the polymer stabilizes the silicate through ionic interactions in pH ranging from 7.0 to 8.5.

**Figure 8:**
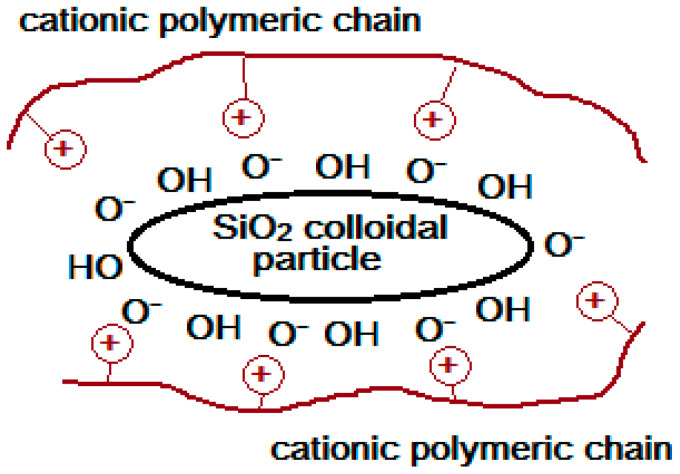
Schematic representation of the interaction of polymer polycation with colloidal silicon dioxide.

**Figure 9:**
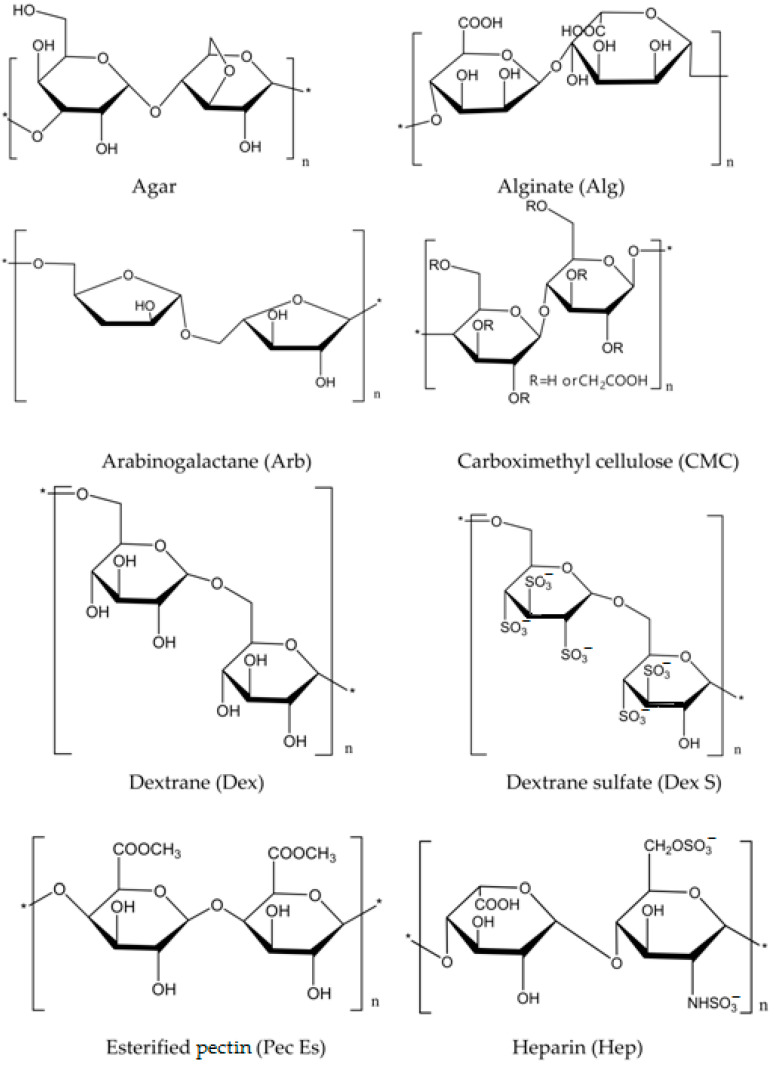
Structural formulas of polysaccharides.

Cationic inulin (CATIN) with varying degrees of substitution (the degree of substitution (DS) is defined as the average number of cationic groups per monomer) stabilizes colloidal silica particles, preventing the formation of polymer silica molecules (Figure 8), and the effectiveness of the inhibition of polymerization depends on DS. Therefore, CATIN DS = 0.22 40 ppm can stabilize 275 ppm silicate, while CATIN DS = 0.86 and 1.28 can stabilize ~340 ppm silicate. It has been noted that increases in the dosage of CATIN have not lead to increases in efficiency, but, on the contrary, to decreases [98]. If the polymerization of silicate occurs in the presence of a cationic polymer additive, then a number of competing reactions occur simultaneously: (1) The polymerization of silicate anions through a mechanism that involves the attack of a monodeprotonated silicic acid molecule on a fully protonated silicic acid molecule. In this process, short-lived silicate dimers are formed, which, in turn, continue to interact with each other, forming colloidal silica particles. (2) The stabilization of silicate ions of a colloidal particle is due to the cation–anion interaction with a cationic polymer molecule (Figure 9). (3) Flocculation between a polycationic polymer inhibitor and negatively charged particles of colloidal silicon dioxide, which are formed as a result of the noninhibited polymerization of silicate [97,98]. Neutral polymers, as well as cationic ones, are able to inhibit silicic acid deposits, but, in this case, the mechanism of inhibition is different. Functional groups should contain fragments with electronegative elements (O and N), which are capable of forming hydrogen bonds with the hydroxyl or silanol groups of silicic acid. Examples of neutral polymers include polyvinylpyrrolidone (PVP), polyethylene glycol with a different molar mass (PEG), polyethylene oxazoline, and polyvinylpyridine copolymer with polyethylene glycol methyl ether methacrylate [99,100,101,102]. It was found that the stabilization efficiency of silicic acid increased with the increase in the molecular weight of the PEG polymer, but this phenomenon leveled off at a molecular weight of ~10,000 Da [100]. It has been shown that with increases in polymer concentration, the effectiveness of inhibiting the polymerization of silicic acid decreases, but, on the contrary, with further increases, provokes its polycondensation [102].

## 5. Mechanisms of Inhibition of Scale Formation in the Presence of Polysaccharides

The mechanism of inhibition of scale formation through polysaccharides for deposits commonly found in technological processes is hardly fundamentally different from inhibition through polymer compounds, such as polycarboxylates, polysulfonates, or any other organic additives [75]. The analysis of the results on the morphology of the crystals formed allows us to conclude that polysaccharides, such as carboxymethylinulin (CMI) [26,103,104,105], carboxymethylcellulose (SMS), functionalized cellulose [106,107,108,109,110,111], and functionalized chitosans [112,113,114], are involved in all stages of the crystallization process. It is known that polysaccharides are involved in the process of biomineralization [22]; however, we note that there is no single type of polysaccharide associated with biominerals, but such polysaccharides are mainly hydroxylate, carboxylate, sulfate, or contain a mixture of these functional fragments. It has been proved that polysaccharides, such as chitin, hyaluronic acid, and keratan sulfate, play an important role in the processes of biopolymerization. It has been shown that polysaccharides, mainly consisting of residues of galacturonic, glucuronic, and mannuronic acids [115,116], are strong inhibitors of CaCO_3_ deposition [117], and participate in the formation of crystal morphology [118]. Alginates, linear polysaccharides consisting of monomeric units of mannuronate and guluronate [118], can form complexes with the Ca^2+^ ion and participate in the formation of hydrogels and in the subsequent formation of calcium carbonate deposits on the cell walls of brown algae. The predominance of polysaccharides in biominerals is an incentive for in vitro research to study their effect on the origin and growth of inorganic phases, as well as to develop methods for creating new hybrid materials, often using calcium carbonate as a model system based on an understandable mechanism. Moreover, research in the field of biomineralization is also of practical importance, resulting in new materials for controlling undesirable scale deposition processes in technological systems. Of course, an important stage was the study of the mechanism of crystallization in the presence of polysaccharides. The works [23,75,119] justified the participation of polysaccharides in the various stages of the formation of an ion cluster: the prenucleation cluster, amorphous phase, and metastable crystal.

**Figure 10:**
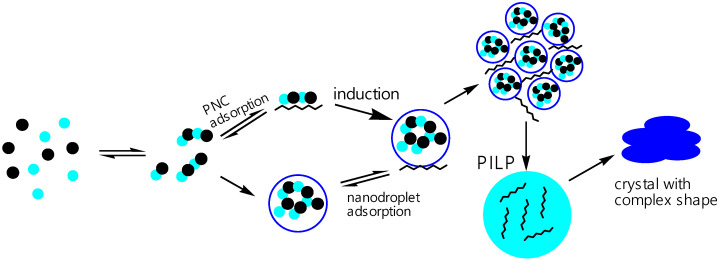
Schematic illustration of the mechanism of formation of crystalline nuclei in accordance with PNC, with polymer adsorption on PNC and nanodroplets (blue circles—cation, black circles—anion). The polymer is integrated into the liquid intermediate product, stabilizing its intermediate states, which can further develop into macroscopic liquid precursors of PILP. The PILP crystal is then transformed into a crystal complex shape [75].

To assess the effect on the crystallization of CaCO_3_, polysaccharides were used as model additives (Figure 9), including agar, alginate (Alg), arabinogalactan (Arb), carboxymethylcellulose (CMC), dextran (Dex), dextran sulfate (Dex S), esterified pectin (Pec Es), and heparin (Hep). In aqueous solutions at a pH of less than 9.5, the carboxylic sulfate dissociated; therefore, polysaccharides, such as alginate, carboxymethyl cellulose, dextran sulfate, and heparin, were shown to have a negative charge. In this regard, complexation with Ca^2+^ could not be ignored. It was found that all polysaccharides with the exception of Pec Es and Hep stabilized the prenucleation cluster (PNC). Nonionized polymers, such as arabinogalactan and agar, could also stabilize the ion cluster, while Pec Es, on the contrary, destabilized the ionic associates in solution. The mechanism of destabilization is not related only to the polymer charge, but it is also necessary to take into account such properties as the chain length and the conformation and branching of the polymer. The crystallization-inhibiting properties are also determined through the time of the formation of ion clusters, so Arb and Dex S practically did not affect the time of the cluster formation, but all other polymers increased the time from 1.16 to 1.66, with Hep reaching 4.35 times. It is assumed that polysaccharides prevent the formation of embryos by stabilizing the colloidal structure of clusters preceding the formation of embryos [120,121]. An important aspect of the processes under consideration is the ability of polysaccharides to form complexes with the Ca^2+^ ion; however, the nature of complexation can be different. For example, Pec Es and a number of other polysaccharides are able to form complexes with a single polymer chain through the electrostatic and donor–acceptor association of poly-D-galacturonate residues, and by crosslinking two parallel chains along the “egg-box” type [122]. It is obvious that polysaccharides (particularly CMC) stabilize the liquid phase of the “polymer-induced” liquid precursor (PILP) of CaCO_3_ [75] (Figure 10).

## 6. Polysaccharides as the Basis of Scale Inhibitors

**Figure 11:**
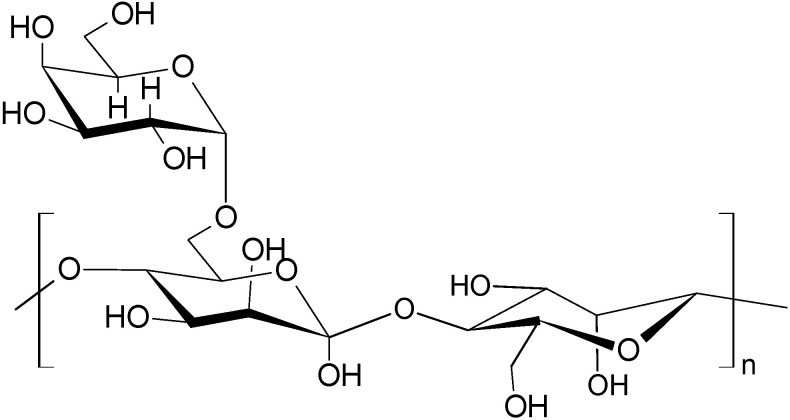
Guar gum (GG).

Current trends in environmental protection and rational use require the development of new highly effective “green” scale inhibitors, the use of which significantly reduces the negative impact on nature and the production of which is not associated with the use of toxic reagents [123]. As shown above, polysaccharides influence the crystallization of calcium scales, in addition to their polymorphic forms, morphology, and particle sizes [22,23,24], all of which stimulate research on the creation of new effective and nontoxic “green” antiscalants to be used in industrial water supply and oilfield reagents as scale deposition inhibitors [13,16,25,26,124]. In particular, guar gum (GG) (Figure 11) was claimed in a patent [125] as the basis of scale deposition inhibitors. Three guar polysaccharides with different molar masses were considered, and it was shown that polymers with an average molar mass of ≈15,000–30,000 Da at a dosage of 10–25 ppm exhibited the highest inhibitory efficiency of CaCO_3_ crystallization. GG with a smaller and with a larger molar mass did not show inhibitory properties. To obtain the required molar mass of GG, it is recommended that H_2_O_2_ be used.

In [126], it was found that degraded guar gel in the composition of liquid waste obtained during hydraulic fracturing entered the reservoir pressure maintenance system. Based on the data on the ionic composition of water from various sources entering the cluster pumping station and calculations of its stability, it was concluded that guar gel with a concentration of 0.05–0.1% at an average molecular weight of (30–40)·10^3^ Da prevented the precipitation of calcium carbonate in the raw water, with an efficiency of 85–90%. As a result, despite the high calcite saturation index, calcite deposits were essentially nonexistent in oil collectors and oil treatment equipment.

**Figure 12:**
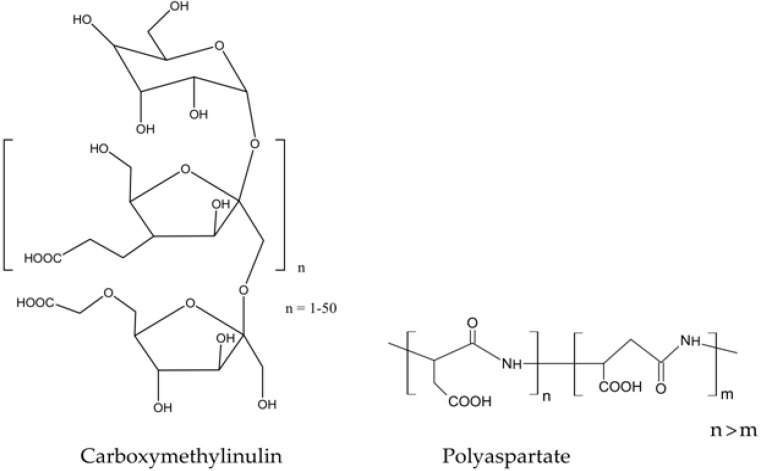
Structural formulas of carboxymethylinulin and polyaspartate.

Carboxymethylinulin (CMI) and polyaspartate (PA) were considered as reagents in the process of injecting an inhibitor into the reservoir [127] instead of a commercially available phosphonate inhibitor based on diethylenetriaminpent (methylene phosphonate) (DETAPF) (Figure 12). In the conducted filtration experiments simulating the injection into the reservoir, the results of CMI and PA were quite satisfactory, despite the fact that DETAPF was still better in terms of the removal time and volumes of protected water.

A detailed study of the mechanism of the inhibition of CaCO_3_ crystallization through the use of carboxyinulin was carried out in [26]. Three samples of carboxyinulin, CMI-15, CMI-20, and CMI-25, were used with varying degrees of substitution of the carboxylate group in the fruit link—1.5, 2.0, and 2.5, respectively. To describe the inhibitory ability of CMI, a kinetic method was used in which the inhibition efficiency was described through Equation (7):

(7) $$\frac{R_{0}}{R_{0}-R_{i}}=\frac{1}{\left(1-b\right)}+\frac{1}{\left(1-b\right)K_{aff}}\cdot \frac{1}{C_{i}}$$

where *K*_aff_ = *k*_ads_/*k*_des_ is the equilibrium constant that is established between the crystalline phase and the inhibitor in the solution, characterizing the affinity of the inhibitor to the crystal surface; *k*_ads_ is the rate constant of inhibitor adsorption; *k*_des_ is the rate constant of the desorption rate; *R*_0_ and *R*_i_ are the rate of growth of the crystalline phase in the absence and in the presence of the inhibitor, respectively; and *b* is the factor limiting the effectiveness of the inhibitor.

Some of the results obtained in this work are presented in Table 3.

The constant *K*_aff_ × 10^7^ determined from Equation (7) was equal to 6.68, 7.94, and 32 L/mol for CMI-15, CMI-20, and CMI-25, respectively. Phosphonate inhibitors, such as oxyethylene diphosphonate and ethylenediaminetetrakismethylene phosphonate, had slightly lower values of 3.7 × 10^6^ and 1 × 10^7^, respectively.

The higher inhibition efficiency of CMI-25 compared with CMI-15 and CMI-20 was associated with a higher affinity for CaCO_3_ crystals. The results of this study indicated that CMI interacted with Ca^2+^ ions on the surface of the forming crystal due to carboxyl groups and due to the size of the macromolecule, making the surface inaccessible for crystal growth. These results were in good agreement with the results of molecular dynamics modeling [104].

Among polysaccharides, active carboxyl groups include, for example, carboxymethyl cellulose and its sodium salt. The availability, environmental friendliness, thermal stability, and biodegradability of Na-carboxymethyl cellulose justify the development of new directions for its application [128,129].

Na-carboxymethylcellulose is one of the most accessible water-soluble polysaccharides, in which the carboxymethyl group is bound to the hydroxyl groups of β-(1→4)-D-glucose monomers [128,129]. NaCMC macromolecules in an aqueous medium have the conformation of elongated tangles, with hypothetically rectilinear sections of 13–18 nm in size (approximately 25 glucose monomers of cellulose), corresponding to the sizes of the formed calcium salt nanoparticles [53,128,130]. Carboxymethylcellulose forms sufficiently stable complexes with the Ca^2+^ ion, which can reduce the precipitation of calcium salts from supersaturated aqueous solutions and prevent the formation of insoluble salts [23].

**Figure 13:**
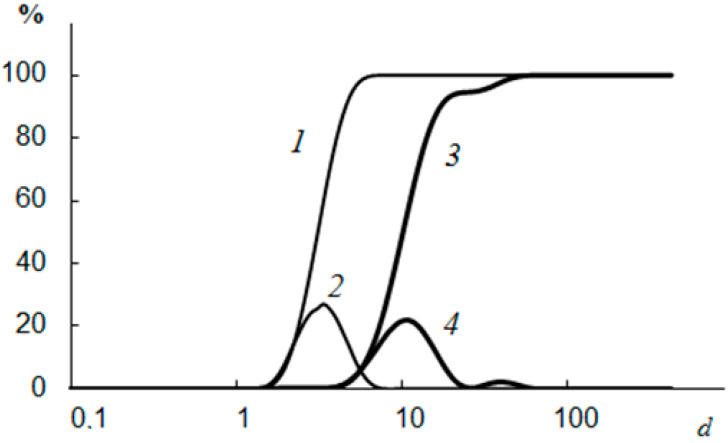
The size of the formed CaCO_3_ crystals without NaCMC (*3*,*4*) and CaCO_3_ crystals in the presence of NaCMC (*1*,*2*); *1* and *3* are integral curves; *d* is the size (µm) [110].

**Figure 14:**
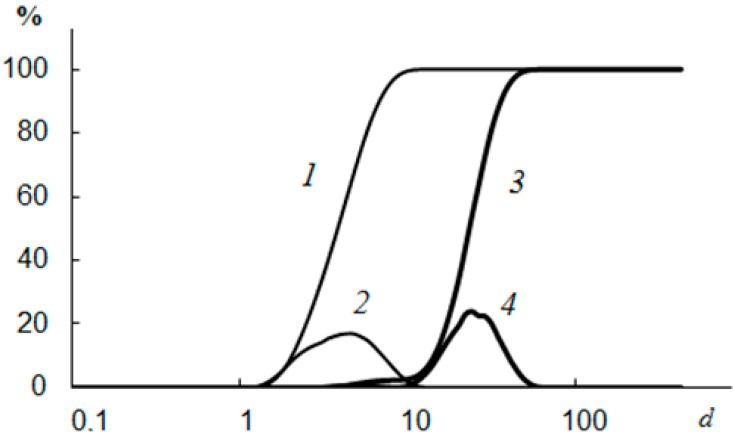
The size of the CaSO_4_ crystals formed without NaCMC (*3*,*4*) and CaSO_4_ crystals in the presence of NaCMC (*1* and *2*); *1* and *3* are integral curves; *d* is the size (µm) [110].

The data obtained using a laser diffraction particle size analyzer on the size distribution of the formed CaCO_3_ and CaSO_4_ crystals showed that in the presence of NaCMC, there was a decrease in the average crystal size by ~7.4 μm for CaCO_3_ and by 17.2 μm for CaSO_4_, as well as displaying the formation of a narrower interval of their distribution, from 1.50 to 7.52 microns for CaCO_3_ and from 1.15 to 11.8 μm for CaSO_4_ (Figure 13 and Figure 14) [110]. The wider range of CaCO_3_ particle size distributions in the presence of NaCMC was probably due to the formation of polysaccharide complexes with CaCO_3_ crystals [131].

**Figure 15:**
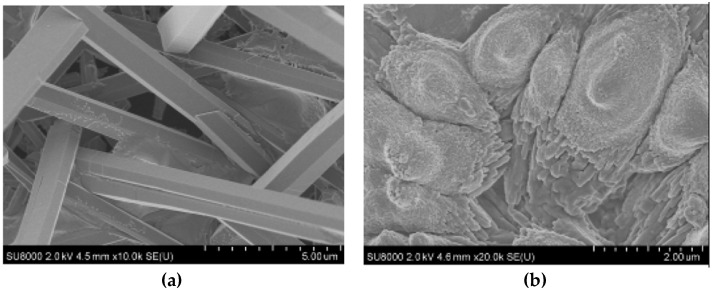
SEM images of CaCO_3_ crystals obtained without (**a**) and in the presence of NaCMC (**b**) [110].

SEM images of CaCO_3_ obtained in the presence of NaCMC indicated the polymorphism of calcium carbonate (Figure 15). The sample obtained in the absence of polysaccharides contained elongated crystals of aragonite, which was the most thermodynamically stable phase under the selected conditions. During the crystallization of calcium carbonate in the presence of NaCMC, the formation of aragonite was practically nonexistent. Crystals are irregularly shaped particles with rounded edges. In the presence of a water-soluble polysaccharide, according to the model of crystallization of calcium carbonate in the presence of active organic additives, the quantitative and qualitative phase composition of the resulting calcium carbonate apparently changes [110].

**Figure 16:**
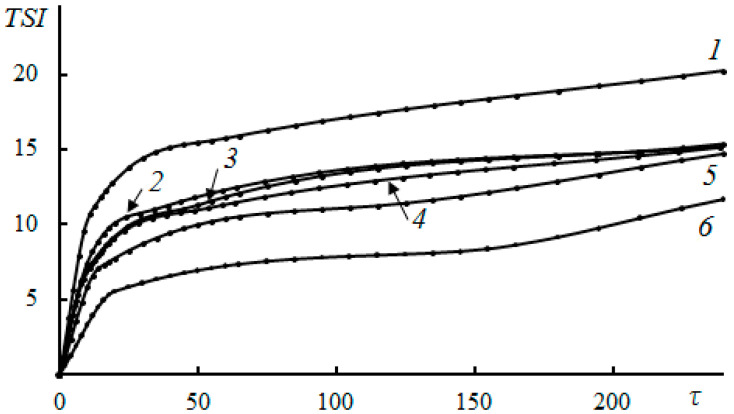
The effect of NaCMC on the process of CaCO_3_ scale deposition at concentrations of 0 (*1*), 10 (*2*), 20 (*3*), 30 (*4*), 50 (*5*), and 100 (*6*) mg/L at 80 °C. *TSI* is the Turbiscan stability index, and τ is the time (min) [110].

**Figure 17:**
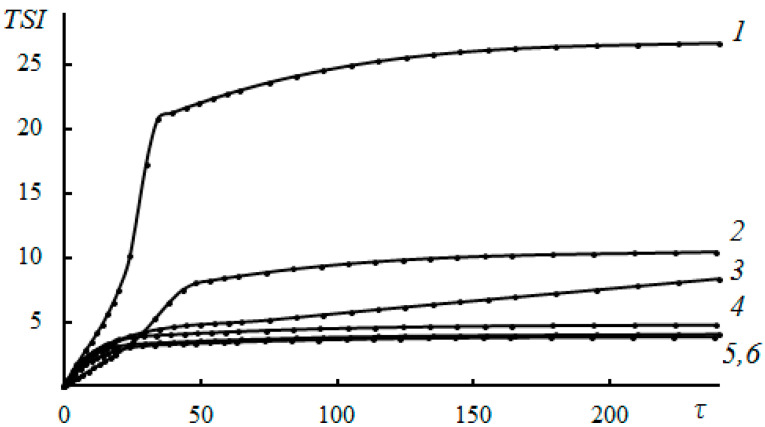
The effect of NaCMC on the process of CaSO_4_ scale deposition at concentrations of 0 (*1*), 10 (*2*), 20 (*3*), 30 (*4*), 50 (*5*), and 100 (*6*) mg/L at 80 °C. *TSI* is the Turbiscan stability index, and τ is the time (min) [110].

The study of NaCMC as an inhibitor of scale deposition through capillary testing at a temperature of 80 °C showed that at a concentration of 30 mg/L, the reagent almost completely inhibited the processes of scale deposition of CaCO_3_ and CaSO_4_, with an effectiveness of ~92–97%. No calcium carbonate crystals were deposited in the capillary during the passage of mineralized water throughout the experiment. The sedimentation stability of time-formed CaCO_3_ and CaSO_4_ in the aqueous phase was studied at NaCMC concentrations of 10, 20, 30, 50, and 100 mg/L at 80 °C. In the absence of NaCMC, a fine suspension of crystals was formed, which, as a result of aggregation and agglomeration [110], settled, and the clarification of the solution was observed (Figure 16 and Figure 17). In this case, the stability index had the highest value of the entire observation period.

**Figure 18:**
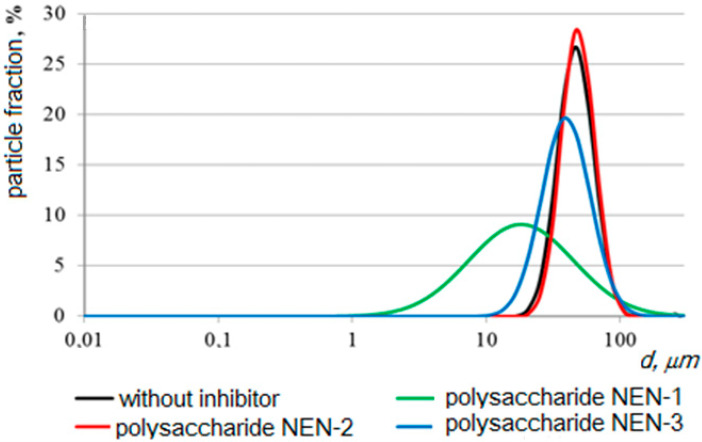
The effect of the polysaccharides NEN-1, NEN-2, and NEN-3 on the size distribution of the formed calcium carbonate crystals.

At concentrations of NaCMC of more than 10 mg/L, the stability index value decreased, indicating an increase in the stability of the dispersed systems of NaCMC-CaCO_3_ and NaCMC-CaSO_4_. The formation and precipitation of CaCO_3_ and CaSO_4_ crystals did not occur within three hours, which indicated the high efficiency of NaCMC as a scale inhibitor. The nature of the interaction between NaCMC and the calcium scale is quite complex. The main reason for the influence of NaCMC on the crystallization of calcium carbonate is likely the specific adsorption of NaCMC on the forming surfaces of calcium carbonate and sulfate, both due to the electrostatic interaction of ionized carboxyl groups with Ca^2+^ ions located on the crystal surface, and the coordination and hydrogen bonds between oxygen atoms and OH groups of D-glucose fragments [23,64,75,122]. This interaction leads to the formation of polymorphic forms without pronounced morphological features and changes in the size of calcium carbonate particles [132,133]. The data obtained indicated the prospect of creating new “green” reagents for oil and gas production based on sodium carboxymethylcellulose scale deposition inhibitors. A capillary test for the effectiveness of the inhibition of scale deposition showed the following series of activities for functionalized polysaccharides: NEN-3 < NEN-1 < NEN-2. At a concentration of 10 mg/L of NEN-2 polysaccharide and 30 mg/L of NEN-1 polysaccharide, there was practically no deposition of calcium carbonate crystals in the capillary when passing mineralized water throughout the experiment. The results in terms of the size distribution of the formed CaCO_3_ crystals under the action of NEN-1, NEN-2, and NEN-3 polysaccharides showed that in the presence of NEN-1, the average crystal size decreased by 32.2 microns, and there was a wider interval of their distribution (Figure 18).

**Figure 19:**
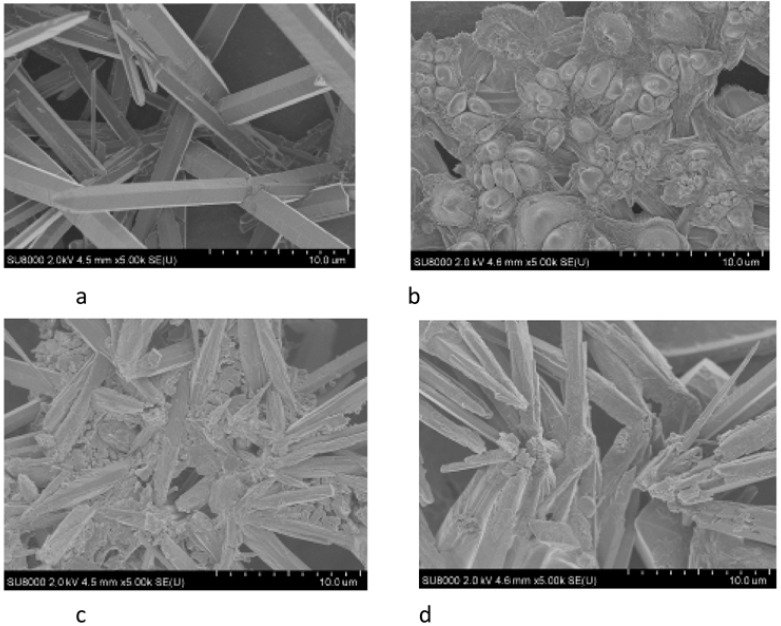
SEM images of calcium carbonate (**a**) and calcium carbonate obtained in the presence of the polysaccharides NEN-1 (**b**), NEN-2 (**c**), and NEN-3 (**d**).

SEM images of calcium carbonate obtained in the presence of various polysaccharides indicated the polymorphism of calcium carbonate (Figure 19). Elongated crystals of aragonite were present in the sample obtained in the absence of polysaccharides. Crystals formed under the action of NEN-1 polysaccharide could be described as more of a “jewelry work of art”.

One of the available water-soluble polysaccharides is arabinogalactan, obtained from larch wood growing in Siberia. The macromolecule of arabinogalactan has a highly branched structure; its main chain consists of galactose links, and the side chains consist of galactose and arabinose links, as well as uronic acids. Arabinogalactan does not exhibit acute toxicity at a dose of 5 g/kg and chronic toxicity at a dose of 500 mg/kg per day, and its physical–chemical properties practically do not change up to 130 °C [134]. Due to their unique spheroidal structure in the aqueous medium, arabinogalactan macromolecules form complexes when interacting with calcium, magnesium, and barium scale, and stabilize suspensions of metal nanoparticles.

The study of arabinogalactan as an inhibitor of scale deposition through capillary testing at a temperature of 80 °C showed that at a concentration of 20 mg/L, the reagent inhibited the process of scale deposition of CaCO_3_, and its effectiveness was ~98% [13,135]. The sizes of the CaCO_3_ crystals formed under the action of arabinogalactan showed that in its presence, a decrease in the average crystal size by ~12 microns was observed. Electronic micrographs of CaCO_3_ in the presence of arabinogalactan indicated the polymorphism of calcium carbonate. Elongated crystals of aragonite were present in the sample obtained in the absence of polysaccharide. During the crystallization of calcium carbonate in the presence of arabinogalactan, there was a complete absence of aragonite, and a change in the size and shape of the crystals formed, which were irregularly shaped particles with rounded edges. This fact was in good agreement with the conclusions regarding the participation of arabinogalactan in the stages of the prenucleation cluster and the stabilization of the “polymer-induced” liquid precursor [23,75].

The effect of water-soluble polysaccharides such as dextrans (cationic, anionic, and nonionic) and soluble starch on the precipitation of calcium carbonate in the model system was discussed in detail in another article [24]. In the absence of additives, the formation of metastable phases of vaterite and amorphous calcium carbonate was observed only in the early stages, whereas, at the end of the process, calcite was formed in the system as the only solid phase. In the presence of starch in the final precipitate, vaterite was detected, while its content increased with the increasing starch concentration, probably due to the inhibition of crystal growth. Noninogenic dextran caused the inhibition of the nucleation of vaterite, which led to the formation of calcite as a prepossessing solid phase during the entire deposition process. The size of the calcite crystals obtained in this way decreased with the increase in the relative molecular weight of neutral dextran. The presence of charged dextrans, cationic or anionic, caused the inhibition of the deposition process as a whole. In the case of anionic dextran, inhibition was probably a consequence of its interaction with Ca^2+^ ions, while cationic dextran was most likely adsorbed electrostatically on the negatively charged surface of calcite and vaterite [24]. The effects of the average molar mass of polysaccharides on the example of branched polysaccharides were considered. The main chain of dextran molecules consists of glucose links connected with an α-1,6 bond, with an average mass of 10,000, 40,000, 70,000, and 500,000 Da. The results of the inhibition of calcium carbonate scale formation showed that the effectiveness decreased with an increasing molecular polymer weight. The introduction of a sulfo- or phosphogroup into the dextran molecule led to an increase in solubility and a 99% inhibition efficiency at a concentration of 5–10 mg/L. It was found that the galactose link increased the inhibitory properties of polysaccharides [24].

**Figure 20:**
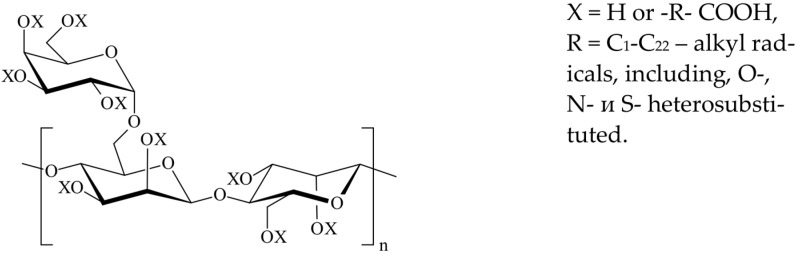
Structural formula of carboxyalkylpolysaccharide.

The revealed general principles and patterns allow us to propose solutions claimed in patents [125,136]. As an inhibitor of the deposition of calcium scale, barium, a product of the depolymerization of polysaccharides, and carboxyalkyl polysaccharide (Figure 20), with a molecular weight of approximately 500,000, containing 0.5 to 3.0 carboxyl groups per sugar fragment, have been proposed, and are used in oil and gas production at concentrations of 10–20 ppm. Polysaccharides available from aloe have been proposed as inhibitors of sedimentation [136], and the construction of polysaccharide molecules presupposes the presence of carboxyl and hydroxyl groups. It is assumed that when these inhibitors are mixed in the form of 5–50% aqueous solutions with a recoverable water–hydrocarbon mixture, they react with divalent alkaline-earth ions of the “egg-box” type.

Polysaccharides of animal origin, such as chitin, are important biomacromolecules of invertebrates that can influence the processes of calcium carbonate biomineralization. Chitin is poorly soluble in aqueous solutions in this regard; hence, water-soluble chitosan and its derivatives are used to study its effect on CaCO_3_ crystallization processes. The properties of chitosan derivatives as compounds affecting the crystallization of calcium carbonate were studied in [77,137]. It was shown that the effect of carboxymethylchitosan [77] with a molar mass of 10^6^ was reduced to the modification of CaCO_3_ crystals due to complexation with Ca^2+^ ions in solution and on the crystal surface. The modified chitosan oligomer [137] exhibited good CaCO_3_ crystallization-inhibiting properties, showing, under comparable conditions, a higher efficacy than other known inhibitors, such as, for example, polyaspartate.

## 7. Conclusions

In recent decades, there have been intense developments in the research field of the inhibition of the crystallization of carbonate, sulfate, phosphate scale, and silicates, as well as improvements in their technological applications for managing scale deposition in industrial water supply processes for use in oil production. In the systematic search for new inhibitors, the study of crystallization processes in their presence and the substantiation of inhibition mechanisms makes it possible to create effective reagents, develop new schemes and application technologies, and determine the limits of the possible effect of inhibitors on scale deposition in production processes. Of particular note is the trend witnessed in the last two decades associated with the development of inhibitors that do not create an additional burden on the existing ecological balance. From a technological point of view, it is important to move towards the development of complex reagents that allow for several problems related to managing complications to be solved at once, for example, for application in oil production. This approach not only makes it possible to solve technological problems, but also opens up opportunities to reduce the use of chemical reagents.

## Figures and Tables

**Table 1:** Solubility of various forms of CaCO_3_ and carbonates at 25 °C.

CaCO_3_ Form	−lg(p*K_sp_*)
Amorphous (monohydrate)	6.4
Ikaite (hexahydrate)	6.62
Vaterite (anhydrous)	7.91
Aragonite (anhydrous)	8.34
Calcite	8.48
Hematite (FeCO_3_)	10.50
MgCO_3_	4.67

**Table 2:** Typical mineral deposits occurring during oil production and on heat exchange equipment.

Sediments	Formula	Nature and Water Source	Conditions of Formation	Basic Crystalline Form	Reference
Calcium carbonate	CaCO_3_	Reservoir water, recycled water	Pressure reduction, temperature increase	Calcite, aragonite, vaterite	[4,5,6]
Calcium sulfate	CaSO_4_.nH_2_O	Formation mineralized water, natural surface water, sea water	Mixing of surface water and underground water with sea water, temperature increase	Gypsum, anhydrite, bassanite	[1,2,3,4,5,6]
Strontium sulfate	SrSO_4_	Formation mineralized water, seawater	Mixing of surface water and underground water with seawater	Celestine	[2,3]
Iron carbonate	FeCO_3_	Reservoir water, seawater, recycled water supply	High content of dissolved CO_2_ in water, corrosion of low-carbon steels	Siderite	[59]
Iron oxides, hydroxides	Fe(OH)_3_, Fe_2_O_3_, Fe_3_O_4_	Surface water	Oxygen-rich air-injected water	Magnetite, hematite	[7]
Iron sulfide	FeS, FeS_2_	Water with a developed biocenosis of sulfate-reducing bacteria	High content of H2S in water, elevated temperature	Pyrite, marcasite, mackinawite, greigite, muscovite	[7]
Sodium chloride	NaCl	Formation mineralized water	Temperature reduction, processes of degassing, removal of water in the form of steam, concentration of solutions	Halite	[56]

**Table 3:** Effect of CMI on CaCO_3_ crystal growth at 25 °C ([Ca^2+^]/[HCO_3_^−^] = 1, *SI* = 4.8).

Polymer	Molar Mass, *M*_w_, g/mol	Inhibitor Concentration, *C*_i_ · 10^9^ mol/L	CaCO_3_ Crystal Growth Rate, *R_i_* · 10^5^, mol/(m^2^.min)	Inhibition Efficiency, %
Blank	-	-	9.16	-
CMI-15	3927	10	4.37	52.3
		15	2.69	70.6
		20	1.97	78.5
		25	1.47	84.0
CMI-20	3524	5	5.22	43.0
		10	2.59	71.7
		15	1.82	80.1
		20	0.47	95.0
CMI-25	10,937	2.5	4.59	49.9
		5	3.66	60.0
		10	1.70	81.4
		15	0.50	94.6

## Data Availability

Not applicable.
